# Export of Precursor tRNA^Ile^ from the Nucleus to the Cytoplasm in Human Cells

**DOI:** 10.1371/journal.pone.0154044

**Published:** 2016-04-21

**Authors:** Min Wei, Xia Zhao, Mi Liu, Meijuan Niu, Elias Seif, Lawrence Kleiman

**Affiliations:** 1 School of Medicine, Nankai University, Tianjin, China; 2 Lady Davis Institute, Jewish General Hospital, McGill University, Canada; 3 State Key Laboratory for Infectious Disease Prevention and Control, National Center for AIDS/STD Control and Prevention, Chinese Center for Disease Control and Prevention, Beijing, China; Institut National de la Santé et de la Recherche Médicale, FRANCE

## Abstract

In the current concept, tRNA maturation in vertebrate cells, including splicing of introns, trimming of 5’ leader and 3’ trailer, and adding of CCA, is thought to occur exclusively in the nucleus. Here we provide evidence to challenge this concept. Unspliced intron-containing precursor tRNA^Ile^ was identified in Human Immunodeficiency Virus type 1 (HIV-1) virions, which are synthesized in the cytoplasm. Northern blot, confocal microscopy and quantitative RT-PCR further verified enrichment of this unspliced tRNA^Ile^ within the cytoplasm in human cells. In addition to containing an intron, the cytoplasmic precursor tRNA^Ile^ also contains a short incompletely processed 5´ leader and a 3´ trailer, which abundance is around 1000 fold higher than the nuclear precursor tRNA^Ile^ with long 5’ leader and long 3’ trailer. *In vitro* data also suggest that the cytoplasmic unspliced end-immature precursor tRNA^Ile^ could be processed by short isoform of RNase Z, but not long isoform of RNase Z. These data suggest that precursor tRNAs could export from the nucleus to the cytoplasm in human cells, instead of be processed only in the nucleus.

## Introduction

Mature transfer RNA (tRNA) plays an important role in protein translation. Its main function is to transfer specific amino acids to growing polypeptide chain in the ribosomal site. In eukaryotes, tRNAs are first transcribed as precursor tRNAs (pre-tRNAs) in the nucleus. Pre-tRNAs undergo a sequential process, including trimming of 5’ leader and 3’ trailer, splicing of introns, modifying nucleosides and adding of CCA, which is termed maturation before tRNA functioning in the cytoplasm for translation [1]. Conventionally, mature tRNAs are thought to solely function in the cytoplasm for translation. However, the discovery of accumulation of mature tRNAs in the nucleus in eukaryotes changes this concept [2]. Two groups have independently shown that mature tRNAs can actively shuttle between the nucleus and the cytoplasm in yeast [2, 3]. The significance of this shuttling is unknown, but implies tRNA quality control, cellular response to nutrient shortage, or cell cycle check-point control in response to DNA damage [4–6].

In eukaryotes, the maturation of pre-tRNAs is conventionally thought to occur in the nucleus [7]. This concept was challenged in yeast. Splicing of intron-containing pre-tRNAs depends on the tRNA splicing endonuclease. Previous studies showed that in yeast, tRNA splicing endonuclease localizes on the surface of mitochondria, and pre-tRNAs export from the nucleus to the cytoplasm for splicing [8]. Intriguingly, the spliced pre-tRNAs are imported to the nucleus for modification of m^1^G, and even re-exported to the cytoplasm for synthesis of wybutosine (yW) [9]. Thus, the shuttling of tRNAs between nucleus and cytoplasm does not only exist in the mature tRNAs, but also in the immature tRNAs in yeast.

However, the shuttling between nucleus and cytoplasm for pre-tRNA maturation in vertebrate cells seems unlikely, since tRNA splicing endonulease SEN, 5’ processing enzyme RNase P, and 3’ processing enzyme RNase Z localize in the nucleus [1, 5]. Thus, the maturation of pre-tRNAs in vertebrate cells is still thought to occur in the nucleus. Nevertheless, here we show evidence for the export of pre-tRNAs from the nucleus to the cytoplasm in human cells.

Human immunodeficiency virus type 1 (HIV-1), the pathogen of AIDS, selectively packages primer tRNA^Lys3^ to initiate the reverse transcription [10]. Selective packaging of tRNAs refers to the relative tRNA percentage increase from the cytoplasm to the virus. For example, the relative abundance of tRNA^Lys^ (which includes both primer tRNA^Lys3^ and major tRNA^Lys^ isoacceptors tRNA^Lys1^ and tRNA^Lys2^, in short tRNA^Lys1,2^) changes from 4.5% in the cytoplasm to 35% in HIV-1 virions produced from human HEK293T cells, indicating an eightfold increase [10, 11]. Recent microarray data revealed that tRNA^Lys^, as well as tRNA^Asn^ and tRNA^Ile^, are all selectively packaged into HIV-1 virions [12]. In this study, surprisingly, we found that tRNA^Ile^ with or without intron were both identified in the virions. Subsequent Northern blot, confocal microscopy and quantitative RT-PCR data further verified enrichment of this unspliced intron-containing tRNA^Ile^ in the cytoplasm. The abundance of this cytoplasmic pre-tRNA^Ile^ is even much higher than the nuclear one. Furthermore, the cytoplasmic unspliced pre-tRNA^Ile^ is end-immature, and contains short 5’ leader and short 3’ trailer sequences. Taken together, these data suggest that the export of pre-tRNAs from the nucleus to the cytoplasm for tRNA maturation is not only in yeast, but also in vertebrate cells.

## Materials and Methods

### 2D-PAGE and Northern Blot Analysis

Two dimensional polyacrylamide gel electrophoresis (2D-PAGE) was performed as previously described [12, 13]. Briefly, purified HIV-1 virions were collected. The total HIV-1 viral RNA were then extracted with Trizol (Invitrogen), and 3’ end labeled with radioisotope [^32^P]pCp, and ran 10% polyacrylamide gel for 10 hours. We then cut the gel with strong radioactive signals, rotated the gel 90 degrees, embedded into 20% polyacrylamide gel and ran it for another 24 hours at 4°C cold room. To identify the packaging of tRNA^Ile^, we cut the 2D-PAGE gel into small pieces, labeled, dissolved each of them in gel elution buffer (20mM Tris-HCl pH 7.5, 5mM EDTA, 400mM sodium acetate), and precipitated with ethanol. The pellet was dissolved in RNase-free water and used as a template for RT-PCR.

For isolation of nuclear and cytoplasmic RNAs, cytoplasmic supernatant was separated from nuclear pellet after lysis of cytoplasmic membrane with buffer (10 mM Hepes pH 7.6, 10 mM NaCl, 3 mM CaCl_2_, 0.5% NP-40, and 400 U/ml RNase inhibitor). The nuclear pellet was washed once with the same buffer. The cytoplasmic supernatant, nuclear pellet, and total cell pellet were subjected to RNA isolation with *mir*Vana^™^ miRNA isolation kit (Ambion). The purified RNA was then ran 10% polyacrylamide gel and hybridized with [^32^P]-labeled DNA probes as indicated in Northern blot analysis (S1 Table).

### Microscopy

Human HEK293T cells (ATCC, CRL-11268, bought from ATCC in November, 2008) were cultured on glass coverslips and transfected with HIV-1. After 48 hours transfection, the cells were fixed in 4% paraformaldehyde for 5 minutes and followed by cold methanol at -20°C for 15 minutes. FISH (Fluorescence in situ hybridization) was performed using probe 5’ Fluorescein-TGCTCACAGGTG-CACTGTCT 3’ (Invitrogen). The cells were stained with or without antibody anti-Gag, Alexa-Fluor-594 labeled secondary antibody (Invitrogen) and DAPI, and examined by Zeiss confocal microscope.

### 5’ RACE and 3’ RACE

Poly A tailing kit (Applied Biosystems), 5’ RACE and 3’ RACE kits (Invitrogen) were used as following the manuscripts. PCR products were cloned into pJET vector (CloneJET PCR cloning Kit, MBI Fermentas) for sequencing. Quantitative RT-PCR was performed using SuperScript II Reverse Transcriptase (Invitrogen) and QuantiTect SYBR Green PCR kit (Cat. No. 204143, Qiagen). Primer sequences were listed in S1 Table. tRNA was transcribed *in vitro* using MEGAscript T7 high yield transcription kit (AM1334, Ambion).

### Purification of RNase ZS

HEK293E cells stably expressing Epstein-Barr virus nuclear antigen-1 were obtained from Yves Durocher (Biotechnology Research Institute, Montreal, Canada), grown in F17 medium (Invitrogen) supplemented with 2 mM L-glutamine and 0.1% Pluronic F-68 (GIBCO), and transfected by linear polyethylenimine (PEI; pH 7.0) (Polysciences Inc.). Human RNase ZS cDNA was cloned into pTT5-Sh5 vector and purified in HEK293E cells as previously described [14].

### 3’ Processing by RNase ZS and RNase ZL *In Vitro*

The assay was performed as previously described [15]. Briefly, *in vitro* transcribed tRNAs were incorporated [^32^P]-UTP (Perkin Elmer). The 3’ tRNA processing assay was carried out in the buffer containing 10mM Tris-HCl (pH 7.5), 1.5mM DTT and 3.2 mM MgCl_2_ at 37°C for 10 minutes. The reaction products were then analyzed in 10% polyacrylamide gel.

## Results

### Identification of Intron-Containing tRNA^Ile^ in HIV-1 Virions

**Fig 1:**
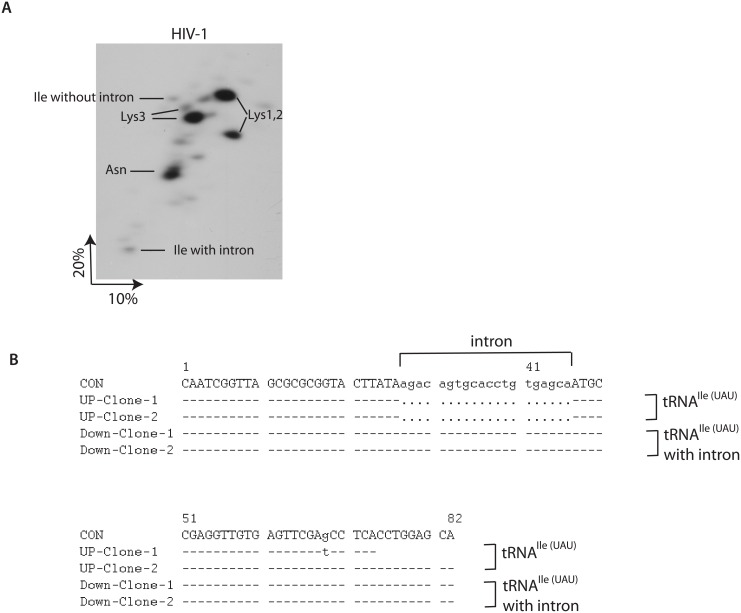
Two dimensional polyacrylamide gel electrophoresis (2D-PAGE) analysis of low molecular weight RNA from HIV-1. (**A**) 2D-PAGE pattern. tRNA^Lys3^, tRNA^Lys1,2^, tRNA^Asn^ are indicated. tRNA^Ile(UAU)^ with and without intron newly identified by RT-PCR and sequencing are also indicated. Sequences of two clones are listed in panel B. (**B**) Sequence alignment of tRNA^Ile(UAU)^. Con represents the consensus of tRNA^Ile(UAU)^. The intron sequences are in small letters. Dash “-” stands for the same sequence as the consensus. Dot “.” stands for the sequence deletion.

Extracellular HIV-1 virions released from human HEK293T cells were purified by ultracentrifugation through sucrose gradients. Viral RNA was then extracted by Trizol reagent and analyzed by two dimensional polyacrylamide gel electrophoresis (2D-PAGE). Only low molecular weight RNA can enter into the gel and results are showed in Fig 1A. tRNA^Lys^ and tRNA^Asn^ were both identified by microarray assay and sequencing in 2D-PAGE analysis previously [12, 16]. Microarray data also indicated that another unexpected tRNA^Ile^ is also selectively packaged into HIV-1 virions [12]. To confirm the packaging of tRNA^Ile^, we cut the 2D-PAGE gel into small pieces, labeled and dissolved each of them in gel elution buffer. RT-PCR and sequencing were followed. tRNA^Ile^ was identified in the upper part of 2D-PAGE as indicated and the sequences of two clones are listed (Fig 1A and 1B). Surprisingly, intron-containing tRNA^Ile^ was also identified in the lower part of 2D-PAGE gel (Fig 1A and 1B). Because the immature intron-containing tRNA^Ile^ is longer than the mature one, it runs slower in 2D-PAGE analysis. Intron-containing tRNA^Ile^ is thought to be processed in the nucleus in human cells, and it is totally unanticipated to be found in HIV-1 virions. Since only tRNA^Ile(UAU)^ (intron sequence: agacagtgcacctgtgagca) (Chr6.tRNA63-IleUAU) was found in HIV-1 virions and other tRNA^Ile(UAU)^ were not, we focused on it in the following study and the following probes and primers were designed to the human Chromosome6-tRNA63-IleUAU sequence.

Like tRNA^Lys^, tRNA^Ile^ is selectively packaged into HIV-1 virions, from 0.6% in HEK293T cells to 4% in the virions, indicating a sevenfold increase relative to other tRNAs [12]. Thus, the identification of intron-containing tRNA^Ile(UAU)^ in the virions can exclude the possibility of leak from the cells.

### Enrichment of the Intron-Containing tRNA^Ile(UAU)^ in the Cytoplasm in Human Cells

**Fig 2:**
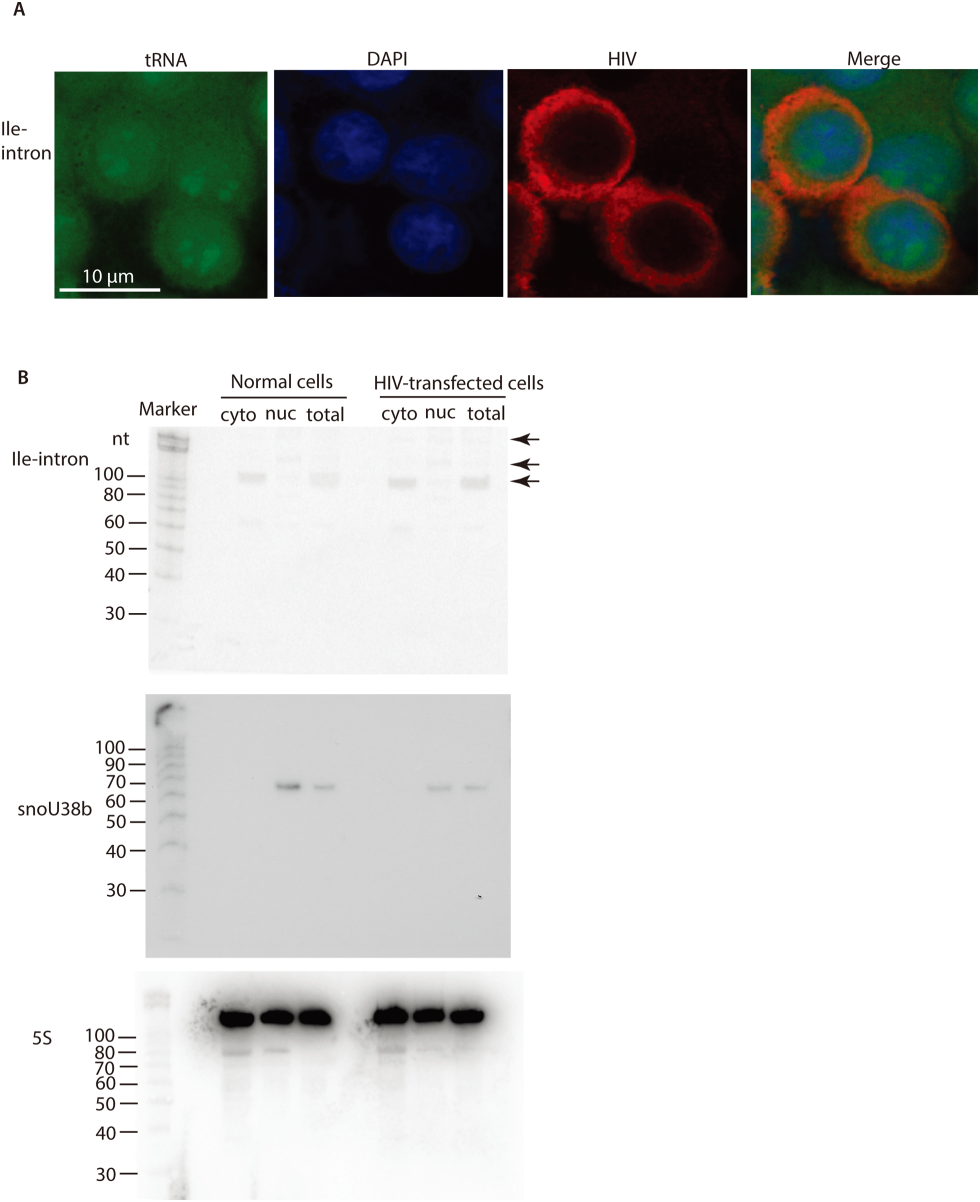
Confocal microscopy and Northern blot analysis. (**A**) Representative immunofluorescence images of cells. Human HEK293T cells were fixed after 48 hours transfection of HIV-1 and stained with a FITC-tagged probe complimentary to the intron of tRNA^Ile(UAU)^ (5’ Fluorescein-TGCTCACAGGTG CACTGTCT 3’, Invitrogen) in FISH as shown in the left panel (green). Nucleus (purple) was stained by DAPI in the left middle panel. HIV-1 Gag (red) was stained by anti-Gag antibody and fluorophore Alexa-Fluor-594 labeled secondary antibody (Invitrogen), as shown in the right middle panel. The pictures were merged in the right panel. (**B**) Northern blot analysis of cellular total RNA, cytoplasmic (cyto) RNA and nuclear (nuc) RNA with or without HIV-1 transfection. 5S RNA was used as a loading control (lower panel) and snoU38B was used as a nuclear marker (middle panel). The intron-containing tRNA^Ile(UAU)^ species are indicated by the arrows (upper panel). The probe is complementary to the intron, as following sequence 5' TGCTCACAGGTGCACTGTCT 3'.

Confocal microscopy was applied to localize the intron-containing tRNA^Ile(UAU)^ in human cells. In a fluorescence in situ hybridization (FISH) assay, fixed cells were hybridized with a FITC-tagged probe complementary to the intron of tRNA^Ile(UAU)^ (Fig 2A). Simultaneously, HIV-1 structural protein Gag was also stained and examined by confocal microscopy (Fig 2A). Much research of HIV-1 has provided evidence that Gag molecules are synthesized in the cytoplasm, assembled on the plasma membrane, from where they subsequently bud into extracellular lumen [17]. HIV-1 Gag molecules are present in the cytoplasm, as showed red color in the right middle panel of Fig 2A. The pictures showed that the intron-containing tRNA^Ile(UAU)^ clustered in some areas of the nucleus, which could be nucleolus. While, the intron-containing tRNA^Ile(UAU)^ was also ubiquitous in the cytoplasm (Fig 2A). In contrast, a negative control, hybridizing a non-specific FITC-tagged probe, gave no signal (data unshown). Thus, the FISH data were specific.

We further separated nuclear and cytoplasmic fractions by centrifugation and ran Northern blot. 5S rRNA was used as a loading control and snoU38b was used as a nuclear marker (Fig 2B middle and lower panel). With or without HIV-1 transfection, the pattern of cytoplasmic and nuclear intron-containing tRNA^Ile(UAU)^ was similar (Fig 2B upper panel), which indicated that HIV-1 transfection did not change its distribution. Surprisingly, intron-containing tRNA^Ile(UAU)^ was mainly present in the cytoplasmic fraction. The size of the major electrophoretic band of cytoplasmic intron-containing tRNA^Ile(UAU)^ was ~110 nucleotides (nt). In contrast, the signal for this species was very weak in the nucleus, and there were two additional bands, ~140nt and ~160nt in size, longer than the cytoplasmic intron-containing tRNA^Ile(UAU)^, as arrows indicated (Fig 2B upper panel). It was unexpected that the abundance of cytoplasmic intron-containing 110nt tRNA^Ile(UAU)^ is even higher than the nuclear one, either.

A reasonable explanation for the identification of intron-containing tRNA^Ile(UAU)^ in the extracellular HIV-1 virions was its presence in the cytoplasm. Then, how do we explain the enrichment of intron-containing tRNA^Ile(UAU)^ in the cytoplasm? This enrichment could be explained by the leak from the nucleus. However, leaking usually occurs from high concentration to low concentration, but does not in an opposite way. Thus, the enrichment of intron-containing tRNA^Ile(UAU)^ in the cytoplasm could be explained by energy-dependent active transport from the nucleus to the cytoplasm. Since the sequence of intron-containing tRNA^Ile(UAU)^ is nuclear-encoded tRNA, instead of mitochondrial-encoded, the enrichment in the cytoplasm excludes the possibility of mitochondrial source.

### Identification of 5’ and 3’ end of the Intron-Containing tRNA^Ile(UAU)^

**Fig 3:**
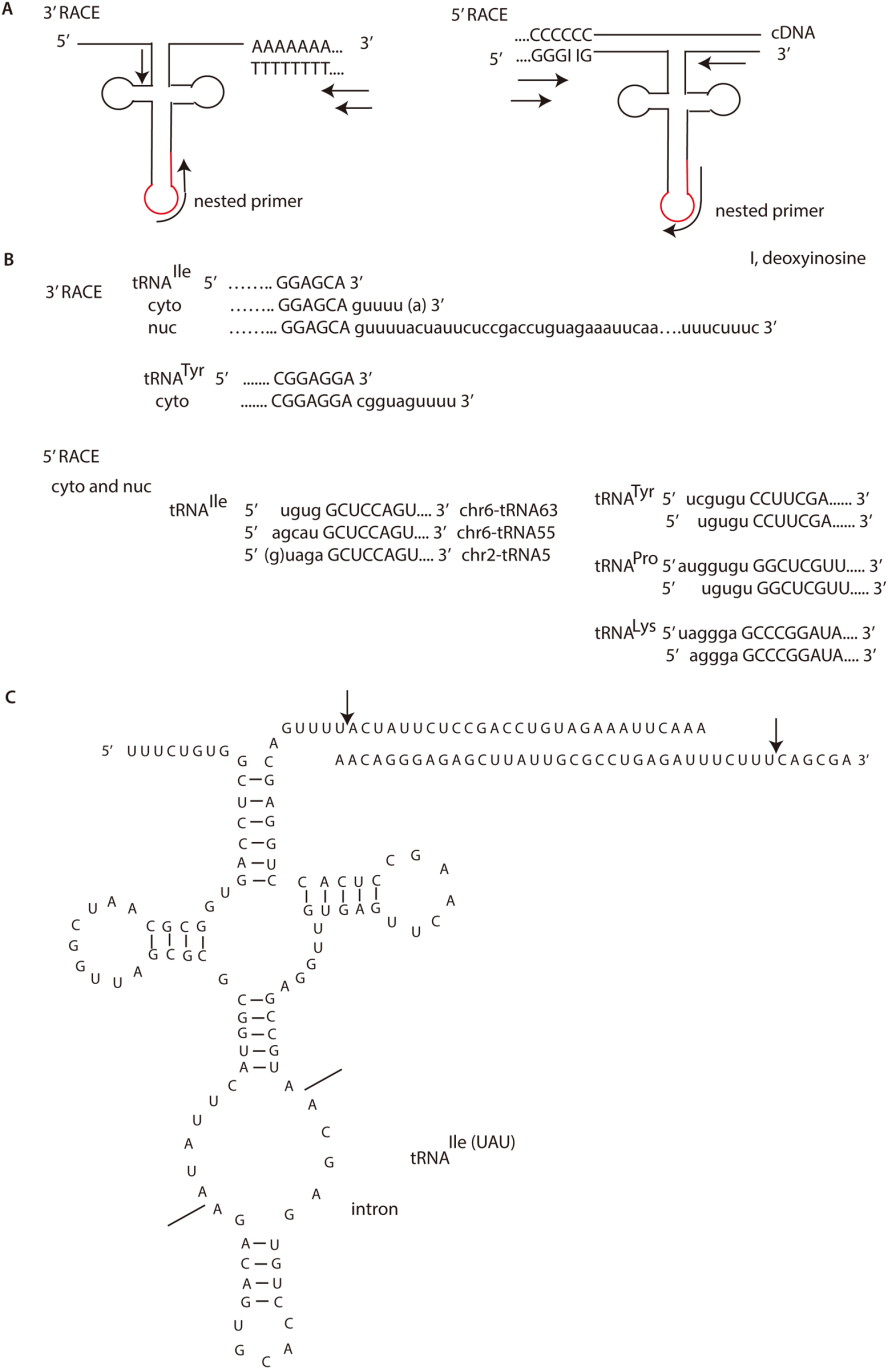
3’ rapid amplification of cDNA ends (RACE) and 5’ RACE analysis. (**A**) Schematic diagram of strategy for 3’ RACE (left panel) and 5’ RACE (right panel). Red curve represents the intron of pre-tRNA^Ile(UAU)^. (**B**) Results of 3’ RACE and 5’ RACE of pre-tRNA^Ile(UAU)^, pre-tRNA^Lys^, pre-tRNA^Pro^, pre-tRNA^Tyr^ from the nucleus (nuc) and the cytoplasm (cyto). 5’ leaders or 3’ trailers are in small letters. (**C**) The secondary structure of the intron-containing tRNA^Ile(UAU)^ (Chr6-tRNA63) with the long 3’ trailer.

Next we tried to get the full length of the intron-containing tRNA^Ile(UAU)^ via developing a strategy to identify its 5’ and 3’ end (5’ rapid amplification of cDNA end, RACE kit (Invitrogen) and 3’ RACE kit). For 3’ RACE, we first added poly A using Poly A tailing kit (Applied Biosystems), and followed the manuscript of 3’ RACE kit (Invitrogen). PCR primer design for 3’ RACE and 5’ RACE is illustrated in Fig 3A, and nested primers are complementary to the intron, which could distinguish between the mature and immature tRNA^Ile(UAU)^. PCR products were cloned into vector pJET (CloneJET PCR cloning Kit, MBI Fermentas) for sequencing. More than a dozen of clones from the cytoplasmic intron-containing tRNA^Ile(UAU)^ were found to bear a short trailer at 3’ termini ending at “5’ guuuu(a) 3’” (Fig 3B). We were not sure the last “a” was from the sequence or from ploy A tailing, so we placed it in parentheses. Another tRNA^Ile(UAU)^ with the intron “5’ cagcagt…3’” was also found to end at “5’ agacccuuu 3’” (Chr2.tRNA5-IleUAU). We also examined another intron-containing tRNA—tRNA^Tyr(GUA)^ (Chr6.tRNA16-TyrGUA). Similarly, the intron-containing tRNA^Tyr(GUA)^ in the cytoplasm also possesses a short 3’ trailer ending at “5’ cgguaguuuu 3’”. Because the following letter after the short 3’ trailer sequence should be “g” according to the human genome, and was impossible to be added by poly A tailing kit, we were sure it ends at “5’ cgguaguuuu 3’” (Fig 3B). We also checked the nuclear 3’ end of intron-containing tRNA^Ile(UAU)^ (Chr6.tRNA63-IleUAU). Some clones with “5’guuuu(a) 3’” were found, but a longer 3’ trailer sequences of ~67 nt until “5’ uuucuuu 3’”, or “5’ uuucu 3’”, or “5’ uuucuuuc 3’” were definitely found in a dozen of clones (Fig 3B and 3C). Other clones with a longer 3’ trailer sequence of 50 nt until “5’ uuauu 3’” were found as well (Fig 3C).

We next examined the 5’ end of pre-tRNAs. The intron-containing tRNA^Ile(UAU)^ (Chr6.tRNA63-IleUAU) had a short 5’ leader “5’ ugug 3’” (Fig 3B). Some clones of 5’ end of intron-containing tRNA^Ile(UAU)^ in other chromosomes were also found to be with short leader “5’ agcau 3’” (Chr6.tRNA55-IleUAU), “5’ (g)uaga 3’” (Chr2.tRNA5-IleUAU) (Fig 3B). Other pre-tRNAs, for example, pre-tRNA^Pro^, pre-tRNA^Lys^, pre-tRNA^Tyr^, were tested as well. It was unanticipated that all of the above mentioned pre-tRNAs possess short 5’ leader both from the nucleus and the cytoplasm (Fig 3B). 5’ RACE never found clones with long 5’ leaders both in the nucleus and the cytoplasm in sequencing dozens of clones.

### Qualitative and Quantitative RT-PCR Analysis of Intron-Containing tRNA^Ile(UAU)^

**Fig 4:**
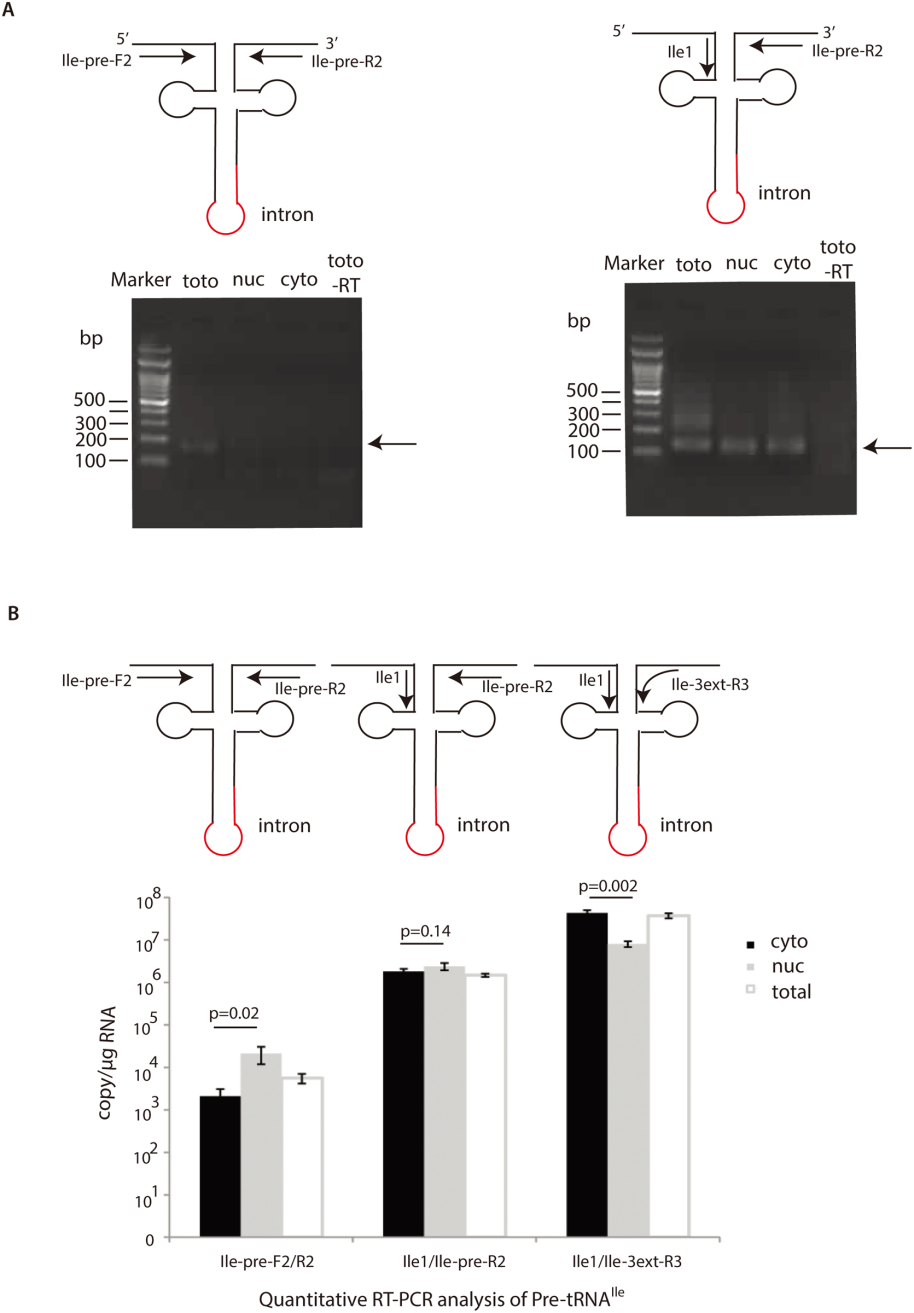
Qualitative (A) and quantitative RT-PCR (B) analysis of the precursor tRNA^Ile(UAU)^. (**A**) Primer name and design are illustrated in the upper panel, and the results of agarose gel electrophoresis are showed in the lower panel. The PCR products are indicated by arrows. Marker, DNA marker; toto, total cell RNA sample; cyto, cytoplasm; nuc, nucleus; total-RT, total RNA sample without reverse transcriptase in reverse transcription. (**B**) Quantitative RT-PCR results. Primer name and design are showed in the upper panel, and the corresponding results are showed in the lower panel. Statistical analysis was conducted between the cytoplasm and the nucleus using two-tail Student T test.

The above data clearly show that the cytoplasmic intron-containing tRNA^Ile(UAU)^, and other pre-tRNAs possess short 5’ leader and 3’ trailer sequences. Next, using RT-PCR, we qualitatively and quantitatively analyzed the intron-containing tRNA^Ile(UAU)^ with elaborate primer design (Fig 4A and 4B). To test whether the RNA samples were contaminated by genomic DNA, we set the negative control containing RNA samples and primers, but, lack of reverse transcriptase. The result was negative (Fig 4B), indicating that RNA sample was clean and RT-PCR data was reliable. In quantitative RT-PCR, we used a plasmid containing the sequence of tRNA^Ile(UAU)^ as a standard. The true copy number of standard was calculated by the concentration of the plasmid and the formula that 1μg 1000bp DNA was equal to 9.1x10^11^ molecules. We found that the nuclear intron-containing tRNA^Ile(UAU)^ with the long 5’ leader and long 3’ trailer is ~10^4^ copy/μg RNA (primer set Ile-pre-F2 and Ile-pre-R2), around 10 fold higher than the cytoplasmic one, which difference has statistical significance (two-tail Student T test, P = 0.02). While the nuclear intron-containing tRNA^Ile(UAU)^ with short 5’ leader, but with long 3’ trailer is ~10^6^ copy/μg RNA (primer set Ile1 and Ile-pre-R2), roughly equivalent to the cytoplasmic one (no statistical significance, P = 0.14). In contrast, the nuclear intron-containing tRNA^Ile(UAU)^ with short 5’ leader and short 3’ trailer (primer set Ile1 and Ile-3ext-R3) is ~10^6^ copy/μg RNA, about 10 fold less than the cytoplasmic one, which difference also has statistical significance (two-tail Student T test, P = 0.002) (Fig 4B). The reverse primer Ile-3ext-R3 complementary to short 3’ trailer sequence was carefully designed to ensure not reacting to the mature tRNA^Ile(UAU)^ ending at CCA. The primer was tested negatively to the plasmid containing tRNA^Ile(UAU)^ ending at CCA, arguing against the interference of mature tRNA^Ile(UAU)^ in quantitative RT-PCR. Furthermore, the melting curves of all quantitative PCR products were single peaks, indicating the specificity of PCR reaction. Other concerns are that the primer set of Ile1 and Ile-pre-R2 can apparently recognize both tRNA^Ile(UAU)^ species with the short or long 5' leaders, and with the long 3’ trailers (Fig 4B). The primer set of Ile1 and Ile-3ext-R3 also recognizes anyone of the tRNA^Ile(UAU)^ species with the long or short 5’ leaders, and with the long or short 3’ trailers. Therefore, the quantitative RT-PCR signals are the sum of those multiple tRNA species. Thus, the true copies of particular tRNA^Ile(UAU)^ species are the values, subtracting the other possible tRNA^Ile(UAU)^ species from the resulting values. However, tRNA^Ile(UAU)^ species with the long 5' leaders and long 3’ trailers are far less than that with the short 5’ leaders and short or long 3’ trailers both in the cytoplasm and the nucleus, and the tRNA^Ile(UAU)^ species with the short 5' leader and long 3’ trailer are also much less than that with the short 5’ leader and short 3’ trailer in the cytoplasm. Thus, the comparation between the quantitative RT-PCR values in the cytoplasm and the nucleus still has significance.

The quantitative RT-PCR data also match the Northern blot data (Fig 2B). The intron-containing tRNA^Ile(UAU)^ with long 5’ leader and 3’ trailer is ~10^4^ copy/μg RNA, beyond the detection limit of Northern blot. So it could not be detected by Northern blot. But it was possible to detect the signal of 10^6^ copy/μg RNA sample with longer exposure time in detection. In Northern blot, a ~110 nucleotide (nt) band in the cytoplasm could correspond to the intron-containing pre-tRNA^Ile(UAU)^ species with short 5’ leader and short 3’ trailer, 105 nt from the sequence. The two weaker longer bands of ~140 nt and ~160 nt in length both in the nucleus and cytoplasm might correspond to the long 3’ trailer sequences until “5’ uuauu 3’” and “5’ uuucuuu 3’”, 145 nt and 160 nt respectively in sequence (Fig 2B). The quantity of 110nt and 160nt bands is also in agreement with the quantitative RT-PCR.

### 3’ Processing of tRNA^Ile(UAU)^ by RNase ZS and RNase ZL

**Fig 5:**
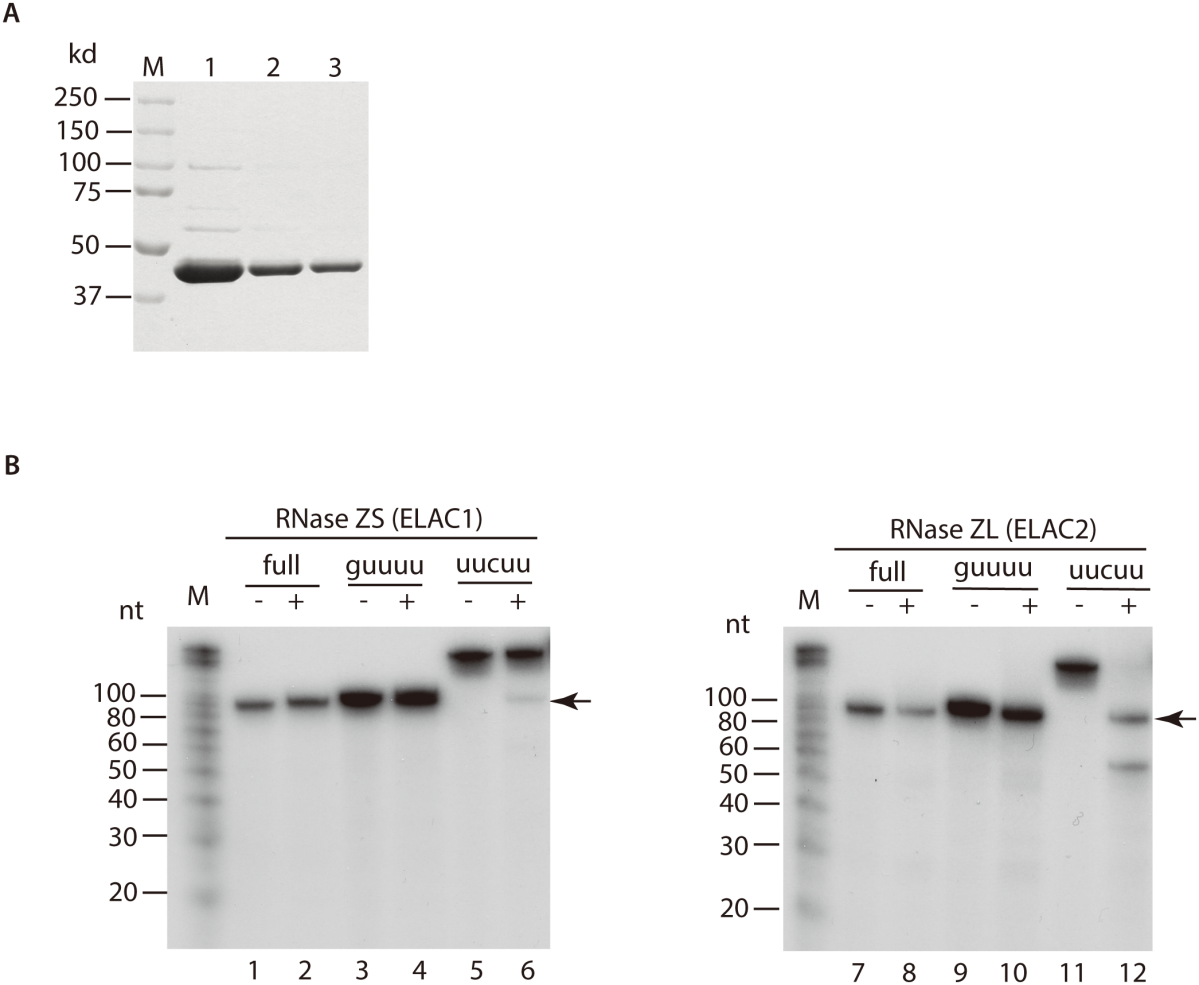
Purification of RNase ZS (A) and *in vitro* 3’ processing of tRNA^Ile(UAU)^ (B) by RNase ZS (RNase Z short, or ELAC1) and RNase ZL (RNase Z long, or ELAC2). (**A**) SDS-PAGE running and Coomassie blue stain of RNase ZS purified from human HEK293E cells. M, protein marker; 1, 2, 3 represents elution 1, 2 and 3. (**B**) *In vitro* 3’ processing with (+) or without (-) RNase ZS or RNase ZL. M, RNA marker. “full” represents full length of intron-containing tRNA^Ile(UAU)^ transcribed *in vitro* without any 5’ leader and 3’ trailer and incorporated radioisotope [^32^P]-UTP. “guuuu” represents the intron-containing tRNA^Ile(UAU)^ with short 3’ trailer until “5’ guuuu 3’”; “uucuu” stands for the intron-containing tRNA^Ile(UAU)^ with long 3’ trailer until “5’ uucuu 3’”.

The above data indicate that the cytoplasmic intron-containing tRNA^Ile(UAU)^ possesses short 5’ leader and short 3’ trailer, ~10^7^ copy/μg RNA. Moreover, the cytoplasmic intron-containing tRNA^Ile(UAU)^ with short 5’ leader and long 3’ trailer is ~10^6^ copy/μg RNA, suggesting that 3’ processing could occur in the cytoplasm. tRNA endonuclease RNase Z is required for tRNA 3’ processing. Human RNase Z comprises two isoforms RNase Z short (RNase ZS, or ELAC1) and RNase Z long (RNase ZL, or ELAC2) [18]. Two-group studies have shown that RNase ZS localizes in the cytoplasm, but RNase ZL resides in the nucleus and mitochondria [19, 20]. Thus, we purified RNase ZS in human HEK293E cells (Fig 5A). The purity of human RNase ZS in elution buffer III was >90% (Fig 5A). Purified RNase ZS and RNase ZL (kindly gift from Dr. Masayuki Nashimoto, Niigata University of Pharmacy and Applied Life Sciences, Niigata, Japan) were used to promote *in vitro* processing of the intron-containing tRNA^Ile(UAU)^. As previously shown, RNase ZL can completely process the tRNA^Ile(UAU)^ with either the short or long 3’ trailer (Fig 5B right panel, please compare lane 9 and 10, lane 11 and 12, and lane 10 and 12) [15]. The band in lane 10 is lower than that in lane 9, and the same level with those in lanes 7 and 8. In contrast, RNase ZS could only incompletely process the 3’ trailer until the “5’ guuuu 3’”, as indicated by the arrow (Fig 5B, left panel, please compare lane 3 and 4, lane 5 and 6, and lane 4 and 6), but cannot remove short 3’ trailer (Fig 5B, please compare lane 3 and 4, lane 9 and 10). This data suggests that cytoplasmic intron-containing tRNA^Ile(UAU)^ with the 3’ trailer terminating at “5’ guuuu 3’” could be probably the product of RNase ZS.

## Discussion

This report clearly shows that the unspliced intron-containing pre-tRNA^Ile(UAU)^ is unexpectedly packaged into HIV-1 virions. Since HIV-1 structural protein Gag molecules are synthesized in the cytoplasm [17], this observation suggests the presence of this pre-tRNA^Ile(UAU)^ in the cytoplasm. Its enrichment in the cytoplasm was further confirmed by confocal microscopy, Northern blot analysis and RT-PCR. An interesting question is followed. Where is the tRNA synthesized, in the nucleus or in the cytoplasm? In eukaryotic cells, a lot of evidence has shown that the bulk of the tRNAs (other than that synthesized in mitochondria) is transcribed by RNA polymerase III in the nucleus [21]. In this study, FISH data showed that intron-containing pre-tRNA^Ile(UAU)^ clusters in some areas of nucleus. Secondly, the nuclear pre-tRNA^Ile(UAU)^ with long 5’ leader and long 3’ trailer is 10 fold higher than the cytoplasmic one. Moreover, 3’ RACE identified long 3’ trailer sequences in the nuclear pre-tRNA^Ile^ as terminating at “5’ uuauu 3’” and “5’ uuucuuu 3’”, which are more likely to be the terminal signal for transcription. Thus, these data still support nuclear source of newly transcribed pre-tRNAs. Therefore, the enrichment of the cytoplasmic intron-containing tRNA^Ile(UAU)^ in human HEK293T cells could be explained by energy-dependent active transport from the nucleus to the cytoplasm. Surprisingly, the cytoplasmic unspliced pre-tRNA^Ile(UAU)^ with short 5’ leader and short 3’ trailer is ~1000 fold higher than the nuclear one with long 5’ leader and long 3’ trailer. The enrichment of pre-tRNAs in the cytoplasm in human cells was also showed in another report, when tRNA-derived RNA fragments (tRFs) were studied [22].

During submission of this manuscript, another paper was published [23]. Using high-throughput sequencing, Eckwahl MJ, et al. identified all small RNAs packaged into another retrovirus Moloney murine leukemia virus (MLV), including precursor tRNAs, small nuclear RNAs, VL30 LTRs, and etc. Like the work reported here for HIV-1, their work confirms that unspliced end-immature precursor tRNA^Ile^ is selectively packaged into MLV and enriched in the cytoplasm. The packaging is dependent on the exportin 5, and pre-tRNA^Ile^ packaging in MLV is decreased 7.4-fold on the depletion of exportin 5. Their work is in agreement with our conclusion that precursor tRNAs can be exported from the nucleus to the cytoplasm.

The limitation of this study is that strong data are missing to support cytoplasmic 3’-end immature pre-tRNA^Ile^ is the product of RNase ZS. Further *in vivo* data and *in vitro* data to other pre-tRNAs are needed. The data in this study do not support the cytoplasmic pre-tRNA^Ile^ with 4U short 3’ trailer is the product of RNA polymerase III, because the latter functions in the nucleus [21].

The cytoplasmic unspliced pre-tRNA^Ile(UAU)^ species are end-immature. So they are not the final products. But, are they intermediates or dead-end products? Interestingly, spliced end-immature pre-tRNA^Ile(UAU)^ was also found in the nucleus in human cells (unshown data), suggesting that cytoplasmic unspliced pre-tRNA^Ile(UAU)^ species are not dead-end products. The techniques used in this study, including RT-PCR and Northern blot, cannot distinguish between on-pathway and off-pathway products, and detect total intermediates.

Takaku H, et al. reported that RNase ZS (ELAC1) could completely process the 3’ terminus of the pre-tRNA^Arg^ *in vitro*, like RNase ZL (ELAC2) [15]. However, their RNase ZS were purified from *E*.*coli* and the RNase ZS in this study was purified from human cells, which may be the reason for our different finding. Previous studies demonstrated that RNase ZS localizes in the cytoplasm, and RNase ZL resides in the nucleus and mitochondria [19]. Localization of RNase ZS matches this study. Thus these data suggest that the unspliced end-immature tRNA^Ile(UAU)^ exports from the nucleus to the cytoplasm probably for 3’ end processing by RNase ZS.

**Fig 6:**
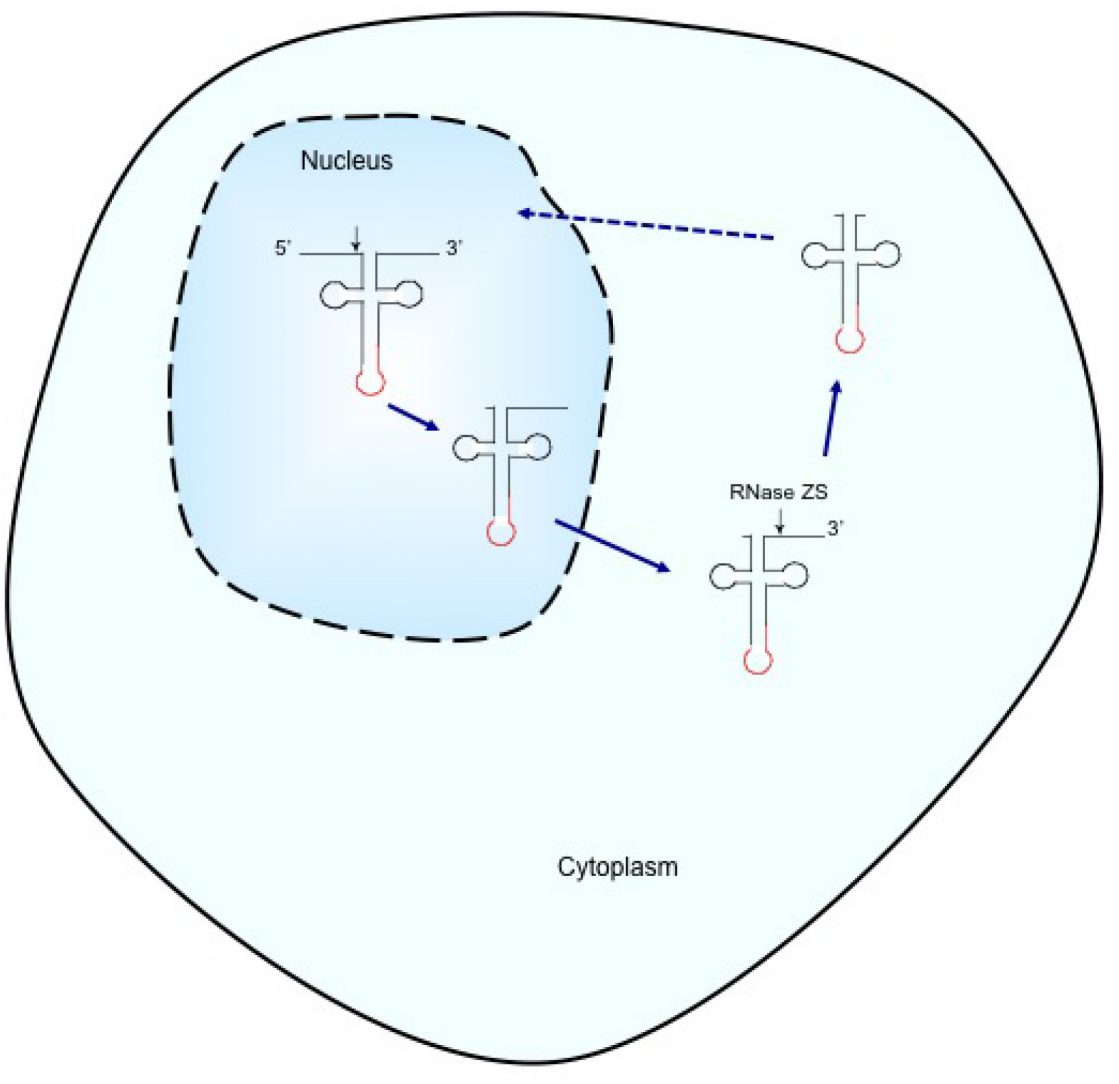
Model of maturation of intron-containing tRNA^Ile(UAU)^ in human cells. The intron-containing pre-tRNAs with long 5’ leaders and 3’ trailers are transcribed in the nucleus and undergo incomplete 5’ processing. They are then exported to the cytoplasm for incomplete 3’ processing by RNase ZS (the solid line with arrows). Retrograde travel of the intron-containing pre-tRNAs with short 5’ leaders and short 3’ trailers is expected from the cytoplasm to the nucleus (the dotted line with an arrow).

The maturation of pre-tRNAs is a sequential process, following the order of 5’ processing, 3’ processing and splicing of introns [24, 25]. This study still agrees with this concept, but raises the possibility of cytoplasmic 3’ processing in human cells. Our data are also in agreement with the report that 5’ leaders of 9 nt or longer severely inhibit the 3’ processing [26]. Based on the data, we propose a model (Fig 6). In human cells, pre-tRNAs are transcribed in the nucleus, and long 5’ leaders are incompletely processed, resulting in short 5’ leaders. These pre-tRNAs are exported to the cytoplasm, where they undergo another incomplete 3’ end cleavage by RNase ZS, resulting in short 3’ trailers. This study raises another unanticipated conclusion that 5’ and 3’ processing of pre-tRNAs are at least two steps or more in human cells, instead of one step as thought before. The short 5’ leaders and 3’ trailers must be removed before tRNA functioning in translation. Next step is splicing. Pre-tRNA splicing, or remove introns from pre-tRNAs, requires the splicing endonuclease SEN, a heterotetrameric complex [25]. Unlike the localization of SEN in mitochondria surface in yeast, human SEN resides in the nucleus [27], and a previous study demonstrated that the pre-tRNA splicing occurs in the nucleus in human cells [28]. In this study, the finding of the unspliced intron-containing tRNA^Ile(UAU)^ in the cytoplasm is absolutely prior to splicing. Indeed, we already found the spliced end-immature pre-tRNA^Ile(UAU)^ in the nucleus (unshown data). Due to the localization of human SEN, RNase P, RNase ZL, and CCA adding enzyme, the cytoplasmic intron-containing pre-tRNA^Ile(UAU)^ has to go back to the nucleus for splicing and final 5’, 3’ processing and CCA addition. Retrograde travel of pre-tRNAs from the cytoplasm to the nucleus was reported in yeast [8]. It is also expected in human pre-tRNAs (Fig 6). However, this study does not show any evidence to support the retrograde of unspliced end-immature tRNA^Ile(UAU)^.

tRNA maturation in vertebrate cells is thought exclusively to occur in the nucleus. This study provides evidence to challenge this concept, and found the export of pre-tRNA from the nucleus to the cytoplasm. We still do not clearly know the function of this export. The maturation of pre-tRNAs in vertebrate cells is so complicated than thought before. Further work is absolutely needed to illustrate the tRNA biogenesis in vertebrate cells.

## Supporting Information

S1 Table Primer sequences for PCR and probe sequences for Northern blot analysis. (DOCX)
