# Supplementary material for: Export of Precursor tRNAIle from the Nucleus to the Cytoplasm in Human Cells
Source: PLoS One. 2016 Apr 21;11(4):e0154044. doi: 10.1371/journal.pone.0154044 (PMC4839721; doi:10.1371/journal.pone.0154044)
Supplement: S1 Table — (DOCX) [file pone.0154044.s001.docx]

**Export of precursor tRNA^Ile^ from the nucleus to the cytoplasm in human cells**

Min Wei, Xia Zhao, Mi Liu, Meijuan Niu, Seif Elias, Lawrence Kleiman

**Supplementary information**

**Table. Primer sequences for PCR and probes for Northern blot analysis**

| Name | Sequences | Usage |
| --- | --- | --- |
| 5S rRNA | 5’ GATCGGGCGCGTTCAGGGTGGTAT 3’ | Probe |
| snoU38b | 5’ AGAACTGGACAAAGTTTTCATCAC 3’ | Probe |
| Ile-UAU | 5' TGCTCACAGGTGCACTGTCT 3' | Probe  5RACE |
| AUAP | 5’ GGCCACGCGTCGACTAGTAC 3’ | RACE |
| 3RACE | 5’ GGCCACGCGTCGACTAGTACTTTTTTTTTTTTTTTTT 3’ | 3RACE |
| 5RACE | 5’ GGCCACGCGTCGACTAGTACGGGIIGGGIIGGGIIG 3’ | 5RACE |
| Ile-intron-F | 5' CCAGTGGCGCAATCGGTTAGCGCGCGGTACTTATAAGACAGT 3' | 3RACE |
| LysTTT-Pre-F1 | 5' GAATGTGCAGGAAGAAGGCTT 3' | PCR |
| LysTTT-Pre-R1 | 5' CAGAATCAGGTACTAAAAGCCACCA 3' | 5RACE |
| LysTTT-Pre-F2 | 5' GAATGTGCAGGAAGAAGGCTTTTG 3' | PCR |
| LysTTT-Pre-R2 | 5' GAATCAGGTACTAAAAGCCACCACTC 3' | 5RACE |
| ProAGG-Pre-F1 | 5' GCCCACAAAGATAACCTTAATGCGTA 3' | PCR |
| ProAGG-Pre-R1 | 5' GAAACGACTTTTACCCTGCACCAA 3' | 5RACE |
| ProAGG-Pre-F2 | 5' GCCCACAAAGATAACCTTAATGCGTAAATC 3' | PCR |
| ProAGG-Pre-R2 | 5' GAAACGACTTTTACCCTGCACCAAAAG 3' | 5RACE |
| Tyr-Pre-F1 | 5' GTCTAAAAAGGACAGCGTTCC 3' | 3RACE |
| Tyr-Pre-R1 | 5' GCTTGGTAGGTCAAAACTACC 3' | 5RACE |
| Name | Sequences | Usage |
| Tyr-Pre-F2 | 5' GTCTAAAAAGGACAGCGTTCCGT 3' | 3RACE |
| Tyr-Pre-R2 | 5' GCTTGGTAGGTCAAAACTACCGT 3' | 5RACE |
| Tyr-intron1 | 5' GGATACCTTGCTTAATGAGC 3' | 3RACE |
| Ile-pre-F1 | 5’ TACCTTTCCAAATCCTTATTATTTCT 3’ | PCR |
| Ile-pre-R1 | 5’ GTTTTTGAATTTCTACAGGTCGGAGA 3’ | PCR |
| Ile-pre-F2 | 5’ TACCTTTCCAAATCCTTATTATTTCTGT 3’ | PCR |
| Ile-pre-R2 | 5’ GTTTTTGAATTTCTACAGGTCGGAGAAT 3’ | PCR |
| Ile-3ext-R3 | 5' GAATAGTAAAACTGCTCCAGGT 3' | PCR |
| Ile1 | 5’ GCTCCAGTGGCGCAATCGGTTA 3’ | PCR |
| TT-ELAC1-BamHI | 5' GATCACGGATCCTCTATGGATGTGACATTCCTG 3' | ELAC1 cloning |
| TT-ELAC1-EcoRV | 5' GGTCGGGATATCTCATTTCTTGATTGGAATGCTTATCAC 3' | ELAC1 cloning |
